# The Influence of OCT3 and MATE2 Genetic Polymorphisms in Poor Response to Metformin in Type 2 Diabetes Mellitus

**DOI:** 10.1002/edm2.486

**Published:** 2024-07-31

**Authors:** Alaa Abd Al‐Hussain Naem, Mona N. Al‐Terehi, Fadhaa Abdulameer Ghafil, Farid S. Ataya, Gaber El‐Saber Batiha, Athanasios Alexiou, Marios Papadakis, Nermeen N. Welson, Najah R. Hadi

**Affiliations:** ^1^ Najaf Health Department Alhakeem General Hospital Najaf Iraq; ^2^ College of Science University of Babylon Babylon Iraq; ^3^ Department of Pharmacology and Therapeutics, Faculty of Medicine University of Kufa Kufa Iraq; ^4^ Department of Biochemistry, College of Science King Saud University Riyadh Saudi Arabia; ^5^ Department of Pharmacology and Therapeutics, Faculty of Veterinary Medicine Damanhour University Damanhour Egypt; ^6^ University Centre for Research & Development, Chandigarh University Mohali India; ^7^ Department of Science and Engineering Novel Global Community Educational Foundation Hebersham New South Wales Australia; ^8^ Department of Research & Development Funogen Athens Greece; ^9^ Department of Research & Development AFNP Med Wien Austria; ^10^ Department of Surgery II University Hospital Witten‐Herdecke, University of Witten‐Herdecke Wuppertal Germany; ^11^ Department of Forensic Medicine and Clinical Toxicology, Faculty of Medicine Beni‐Suef University Beni Suef Egypt

**Keywords:** MATE, metformin response, OCTs, pharmacogentics, soluble carries gene polymorphisms, Type 2 diabetes mellitus

## Abstract

**Background:**

The response of patients with Type 2 diabetes mellitus (T2DM) to metformin may be a variation because of genetic differences in solute carrier (SLC) transporter proteins and other effect factors, which have an important effect on how metformin is processed in the body and its efficiency for glycaemic control.

**Aim:**

This study was conducted to investigate the impact of certain genetic variants of the organic cation transporter genes OCT3 (SLC22A3 rs12194182 and rs8187722) and MATE2 (SLC47A2 rs12943590) and their association with glycaemic parameters in patients with T2DM who respond poorly to metformin.

**Patients and Methods:**

This cross‐sectional study involved 150 Iraqi cases with T2DM who were prescribed a daily dose of (1000 mg/day) metformin for a minimum of 3 months. Various parameters included are as follows: demographic data, glycaemic parameters and three SNPs: rs12943590 variant of SLC47A2, rs12194182 and rs8187722 variant of SLC22A3 using the standard PCR‐sequencing technique.

**Results:**

Thirty‐nine patients (26.17%) were responders, whereas 111 patients (73.82%) could not respond to metformin treatment. Upon analysing the genotypes of the rs12943590 variants of SLC47A2, rs12194182 and rs8187722 SNPs of SLC22A3, the present findings revealed a nonsignificant association of genetic variations in all SNPs with metformin response. SLC47A2 (rs12943590) showed nonsignificant associations of the GG, AA and AG genotyping; SLC22A3 (rs12194182) showed nonsignificant associations of the TT, TC and CC genotyping; and SLC22A3 (rs8187722) showed nonsignificant associations of the AA, CC and AC genotyping between two groups.

**Conclusion:**

Variations in genes SLC22A3 and SLC47A2 did not have a significant role in the response of patients with T2DM to metformin (1000 mg/day).

## Graphical abstract

The genotypes of the rs12943590 variants of SLC47A2 showed no significant variance to the patient's response to metformin. The genotypes of the rs12194182 variants of SLC22A3 showed no significant variance to the patient's response to metformin. The genotypes of the rs8187722 variants of SLC22A3 showed no significant variance to the patient's response to metformin.

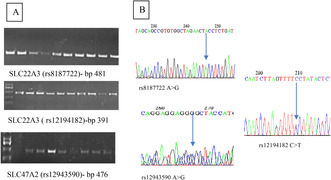

**Summary**

- The genotypes of the rs12943590 variants of SLC47A2 showed no significant variance in the patient's response to metformin.
- The genotypes of the rs12194182 variants of SLC22A3 showed no significant variance in the patient's response to metformin.
- The genotypes of the rs8187722 variants of SLC22A3 showed no significant variance in the patient's response to metformin.
- It is important to note that several factors contribute to the variation in metformin responses including ethnicity and the relationship between genetic factors and the disease itself.

## 1 Introduction

The occurrence of Type 2 diabetes mellitus (T2DM) is on the rise, with estimates from the Centers for Disease Control and Prevention indicating that 11% of adults in the United States aged 20 and older have either been diagnosed with T2DM or have it undiagnosed. Additionally, about 35% of individuals within one age group are facing prediabetic challenges, as determined by their fasting blood glucose or glycated haemoglobin A1c (HbA1c) levels. This represents a significant health concern. Globally, T2DM has become an epidemic, with the number of affected individuals having surged from 108 million in 1980 to a remarkable 422 million in 2014 [1]. Having T2DM puts individuals at a heightened risk of developing serious complications such as vision impairment, kidney dysfunction and even the possibility of lower limb amputations. Furthermore, those with T2DM are 2–4 times more likely to suffer from cardiovascular diseases, including heart attacks and strokes. This underscores the urgent need for effective strategies to prevent and manage T2DM and its associated health risks [2].

Metformin, a pharmaceutical substance related to biguanide, is now a primary pharmacological option for individuals with T2DM [3]. However, despite extensive research efforts, the exact molecular mechanisms responsible for metformin's positive impact on controlling blood sugar levels are still a subject of controversy and limited understanding [4]. Studies, mostly conducted in animal models, have proposed diverse mechanisms and molecules that might contribute to metformin's beneficial effects. These include direct inhibition of mitochondrial action, stimulation of hepatic AMPK (a cellular energy regulator) and changes in glucagon signalling pathways. Nevertheless, the extent to which these mechanisms are responsible for metformin's positive effects on blood sugar control in humans remains unclear [5].

Substantial differences exist in how individuals respond to metformin [6], indicating the potential for more personalised and precise treatment if we gain a deeper understanding of how metformin operates. Research indicates that genetic factors play a role in this variability, offering an opportunity to identify specific genetic factors contributing to these differences through genetic association studies [7].

Pharmacogenetics differs from more classical genetic approaches in that there must be an interaction between genes and drug therapy, as opposed to a more straightforward association with disease. Drug–genome interactions can occur in a number of ways. Pharmacogenetic interactions can result from genetic variation in the primary molecular target of a drug class. One clear example of this is observed in genes related to the drug ADME that stands for absorption, distribution (like OCT1), metabolism and excretion. ADME genes, in general, can influence the effectiveness of numerous drugs, irrespective of the drugs' intended molecular targets [8].

The SLC22A3 gene is expressed in several tissues, such as the brain, muscle, and heart and it produces the organic cation transporter 3 that is responsible for the excretion and elimination of several medications, including metformin. The presence of genetic polymorphisms within this transporter has been associated with significant differences in the pharmacokinetics of metformin and sulfonylureas, which reduce the drug's antidiabetic effectiveness [9, 10]. SLC22A3 is expressed in several tissues, such as the brain, muscle and heart. Several genome‐wide association studies linked SLC22A3 to the risk of prostate cancer and coronary artery disease [11, 12].

The SLC47A2 gene is mainly expressed in renal proximal tubules and it develops multidrug and toxin extrusion (MATE) transporters, which influence the efflux of several hydrophilic organic cations, including metformin. Genetic variations are a crucial factor of a patient's reaction to metformin [13].

Therefore, current study aimed to evaluate the effect of SLC22A3 and SLC47A2 genetic polymorphisms on diversity in metformin response as monotherapy in Iraqi people with T2DM.

## 2 Patients and Methods

### 2.1 Study Design

A cross‐sectional study was held between April 2022 and June 2023, involving a sample of 150 individuals diagnosed with T2DM on the basis of the 2012 American Diabetes Association criteria. These criteria define T2DM using parameters such as HbA1c levels ≥6.5%, fasting plasma glucose (FPG) levels ≥126 mg/dL, 2‐h plasma glucose levels ≥200 mg/dL during an oral glucose tolerance test (OGTT) or random plasma glucose levels ≥200 mg/dL. The study participants were recruited from the diabetes centre at Al‐Sadar Teaching Hospital in Najaf, Iraq. The study is held in accordance with the Helsinki Declaration and informed consents were obtained from all the participants. The study received ethical approval from the Medical Ethics Committee of the Faculty of Medicine at Kufa University.

### 2.2 Study Population

The study population consisted of 150 individuals with T2DM, encompassing both males and females, who had been undergoing a monotherapy regimen of metformin tablets (1000 mg/day) for a minimum of 3 months [14]. These participants fell within the age range of 30–70 years.

The exclusion criteria encompassed patients with significant organ dysfunction, including heart, liver and renal failure, individuals above 70 years of age; those with a BMI exceeding 30 kg/m^2^, pregnant women, patients with chronic gastrointestinal disorders or malabsorption syndrome and individuals concurrently using other oral hypoglycaemic agents (OHAs) or insulin.

According to the glycaemic control, the patients were categorised into two groups on the basis of HbA1c: well‐responders (HbA1c levels <6.5%) and poor responders (HbA1c levels >6.5%).

### 2.3 Data Collection

The data collection process involved the investigator administering a standardised questionnaire to gather demographic and clinical information from patients. This information encompassed their names, ages, body weight and height, duration of illness, medical history, family medical history, dietary habits, sleep patterns and occupations. To calculate the Body Mass Index (BMI), measurements for weight and height were taken. Height measurements were acquired with subjects standing upright, barefoot, with arms at their sides and feet close together. Weight measurements were recorded with patients standing on a scale, wearing lightweight clothing, and without shoes or socks.

BMI was calculated using the formula BMI = weight (in kilograms)/height (in metres squared), and it was used to categorise patients as either normal (BMI < 25 kg/m^2^), overweight (BMI between 25 and 29.9 kg/m^2^) or obese (BMI ≥ 30 kg/m^2^) [15].

The glycaemic control parameters measured include FBG (fasting blood glucose), HbA1c, serum insulin, Homeostasis model assessment for insulin resistance (HOMA‐IR) and insulin sensitivity [16].

### 2.4 Blood Sample Collection

Morning samples of blood were collected from each patient after an overnight fast of 8–12 h. Although patients were seated, about 5 mL of venous blood was drawn using disposable syringes. This blood collection involved distributing 3 mL of blood into tubes containing EDTA and cautiously transferring the remaining 2 mL into serum tubes equipped with separating gel. The blood is stored in EDTA tubes for assessing HbA1c via the immunoassay technique. Blood within the serum tubes was allowed to coagulate at room temperature for around 10–15 min, followed by centrifugation at approximately 3000 × *g* for approximately 3 min. The resulting serum was then stored at a temperature of −80°C until analysis. Serum insulin levels were measured using the BT LAB ELISA kit, following the manufacturer's recommended procedure. Fasting blood glucose was measured utilising the RanDox kit that relies on the PAP enzymatic method for determining glucose levels.

All data were managed using SPSS version 22, with the ANOVA test and *t*‐test used for multiple comparisons and the chi‐square test for the utilisation of non‐numerical variables. Values of ≤0.05 will be considered to be statistically significant.

### 2.5 SNP Genotyping

DNA was obtained from peripheral blood samples using the Favorgen MiniPrep kit, following the manufacturer's instructions. Single nucleotide polymorphisms (SNPs) were screened, and genotyping was performed using the standard PCR method. Specifically, the extracted DNA underwent analysis for the SNPs SLC22A3 rs12194182 and rs8187722, as well as SLC47A2 rs12943590, using custom primers sourced from Macrogen. The sequencing results for these primers are outlined in Table 1.

**TABLE 1:** Primer sequence of study SNPs.

SNPs	Primers sequences	TM°	Product size (bp)
SLC47A2 (rs12943590)	F 5′‐TCAGCTTCCTCCACTCCCTA‐3′	58.1	476
	R 5′‐CTGGACCTTCTCCTGCTGAG‐3′		
SLC22A3 (rs8187722)	5′‐GTGAAACGTGCAGAATGGAA‐3′	54	481
	R 5′‐TGAATTGGCTCTCAAAACTGAA‐3′		
SLC22A3 (rs12194182)	5′‐CACTGACCCAGCTTTATTCAT‐3′	54	391
	R 5′‐CTCCTCCTTCAGCAGTGTCC‐3′		

To ensure optimal PCR conditions, a temperature gradient was initially employed for primer sets. Once the ideal annealing temperature was determined, the PCR mixture was composed of less than 250 ng of template DNA, 400 μM of each dNTP, 12.5 μL of a 1 U GoTaq DNA polymerase buffer, 10 μM of each primer and 3 mM MgCl_2_, with a total reaction volume of 25 μL. Amplification reactions were carried out using a Series thermocycler (Biometra/USA) apparatus. The following program was (Table 2) set in the thermocycler after the identification of the optimum annealing temperature for the amplification of rs12943590 rs12943590, rs12194182 and rs8187722 genes.

**TABLE 2:** Program used for amplification sequences.

Stage	Temperature (°C)	Time (min)	Function	Cycles
rs12943590				
1	95	05:00	Initial denaturation	1
2	95	00:30	Denaturation	35
	58.1	00:30	Primer annealing	
	72	00:30	Template elongation	
3	72	10:00	Final elongation	
4	4	—	Hold Incubation	
rs12194182				
1	95	05:00	Initial denaturation	1
2	95	00:30	Denaturation	35
	54	00:30	Primer annealing	
	72	00:30	Template elongation	
3	72	10:00	Final elongation	
4	4	—	Incubation	Hold
rs8187722				
1	95	05:00	Initial denaturation	1
2	95	00:30	Denaturation	35
	54	00:30	Primer annealing	
	72	00:30	Template elongation	
3	72	10:00	Final elongation	
4	4	—	Incubation	Hold

The amplification products of these genes were observed through agarose gel electrophoresis. A 2.5% agarose gel with 0.5× TBE buffer was employed, and the electrophoresis was conducted at 100 V for a duration of 60 min. After electrophoresis, the bands were made visible by staining them with ethidium bromide and examining them under UV light. To estimate the size of the fragments, a 100 base pair DNA ladder (100–1500 bp) was utilised as a reference marker.

**FIGURE 1:**
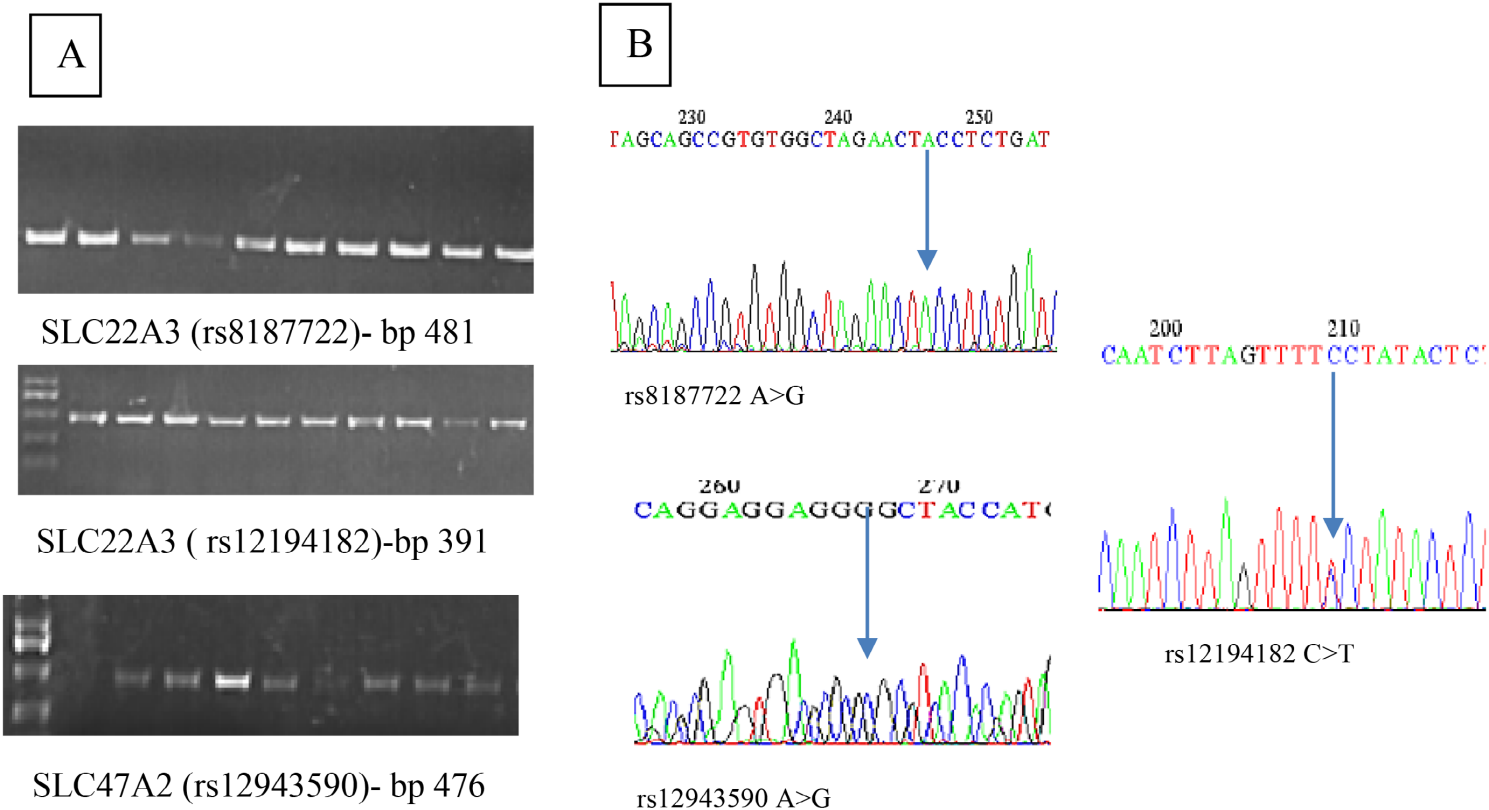
PCR measurements of the studied alleles. (A) Electrophoresis approach. (B) Target SNPs sequencing. rs8187722 A>G, rs12943590 A>G and rs12194182 C>T.

The results of the genetic study were illustrated by DNA electrophoresis, target SNP amplification, and DNA sequencing. The whole genomic DNA concentration and purity ranged 80–156 ng/μL, with purity ranging 1.6–2.2. Electrophoresis showed single sharp bands, as shown in Figure 1. All data were managed using SPSS version 22. Multiple comparisons were conducted using ANOVA and *t*‐tests, whereas non‐numerical variables were assessed using the chi‐square test. The Hardy–Weinberg equilibrium was applied to evaluate all the genes under study. Statistical significance was determined with a *p* value of ≤0.05. The participants were divided into two groups on the basis of their HbA1c percentage: good responders (HbA1c < 6.5%) and poor responders (HbA1c ≥ 6.5%). Allele frequencies were represented as percentages and were statistically analysed using an odd ratio with a 95% confidence interval at *p* < 0.05. Factor analysis is used to confirm the distribution of study samples according to the impacts of glycaemic parameters by reducing variables to two factors.

## 3 Results

About 150 patients with T2DM were included in the study. The patients were classified into two subgroups according to their glycaemic control: poorly controlled diabetics (HbA1c ≥ 6.5%), who were 73.82% (*n* = 111 patients), compared with good glycaemic control (HbA1c ≤ 6.5%), who were 26.17% (*n* = 39 patients) (Table 2). Sociodemographic factors such as BMI, age, education, occupation, gender, presence of other medical conditions and past medical history revealed differences between the two groups. However, these differences were statistically nonsignificant (with *p* ≥ 0.05). On the other hand, there was a notable and statistically significant difference between the two groups in terms of disease duration and the patients' medical histories.

The difference in glycaemic parameters between the good and poor responders is represented in Table 3. The analysis reveals a substantial disparity in mean glycaemic parameters between individuals who responded well and those who responded poorly to metformin treatment. Notably, there were highly significant differences in FBS, HbA1c and HOMA‐IR between these two groups, as indicated by *p* values of 0.000, 0.000 and 0.019, respectively. Although serum insulin levels were observed to be higher in poor respondents compared with good respondents, they were not statistically significant.

**TABLE 3:** Sociodemographic distribution of study group.

Variables	≤6.5 (*n* = 39; 26.17%)	>6.5 (*n* = 111, 73.82%)	*p*
Patients history			
No	18 (45%)	71 (64.54%)	**<0.001**
Yes	22 (55%)	39 (35.45%)	
Age	52.00 ± 1.648	53.90 ± 1.82	0.260
BMI (kg/m^2^)	27.93 ± 0.58	28.01 ± 0.63	0.940
Duration (years)	6.13 ± 0.94	7.94 ± 0.55	0.095
Age category			
<40 years	4 (10%)	7 (6.36%)	0.4631
40–50 years	13 (32.5%)	30 (27.27%)	
>50 years	23 (57.5%)	73 (66.36%)	
Duration category			
<5 years	22 (55%)	38 (34.54%)	**0.038**
5–10 years	8 (20%)	43 (39.09%)	
>10 years	10 (25%)	29 (26.36)	
Education			
Primary	26 (65%)	83 (75.45%)	0.434
Intermediate	12 (30%)	22 (20%)	
University	2 (5%)	5 (4.54%)	
Job			
Yes	12 (30%)	39 (35.45%)	0.532
No	28 (70%)	71 (64.54)	
Drugs (antihypertensive, thyroxin)			
No drugs	19 (47.5%)	62 (56.36%)	0.358
On treatment	21 (52.5%)	48 (43.63%)	
Gender			
Male	10 (25%)	45 (40.90%)	0.0837
Female	30 (75%)	65 (59.09%)	
Another related disease (hypothyroidism, HT)			
Yes	29 (72.5%)	73 (66.36%)	0.519
No	11 (27.5%0	36 (33.63%)	
FBC (mg/dL)	138.7 ± 9.755	248.62 ± 8.843	**<0.001**
HbA1c (%)	6.036 ± 0.070	9.77 ± 0.203	**<0.001**
Insulin (μU/L)	7.148 ± 1.701	9.57 ± 1.26	0.355
HOMA‐IR	45.59 ± 11.55	105.05 ± 14.39	0.019
Insulin sensitivity	1.18 ± 0.08	1.03 ± 0.047	0.587

*Note*: Significance values are indicated in bold.

From Table 4, the results showed SLC47A2 rs12943590 was represented by GG, AA, and AG genotypes, with the major allele GG (25) and the minor allele AA (8), so G>A with allele frequencies of 0.677 and 0.322, respectively, and a genotyping success rate of 32%. SLC22A3 rs12194182 was represented by CC, TT and TC genotypes, with the major allele TT (71) and the minor allele CC (2), so T>C with allele frequencies of 0.859 and 0.140, respectively, and a genotyping success rate of 64%. SLC22A3 rs8187722 was represented by AA, CC and AC genotypes. The rs8187722 has the major allele AA (65) and the minor allele CC (2), so A>C with an allele frequency of 0.781 and 0.218, respectively, and a genotyping success rate of 74.67%. As shown in Table 5, regarding the rs12943590 gene, the GA allele showed a lower mean of HbA1c, FBS, serum insulin and HOMA‐IR than GG and AA but was statistically nonsignificant. The GA allele also has the highest insulin sensitivity.

**TABLE 4:** Genotypes distribution of study groups (chi‐square, *p* < 0.05).

SNPs	*n*	Allele frequency
rs12943590		
GG	25	G = 0.677
AG	15	A = 0.322
AA	8	
rs12194182		
TT	71	T = 0.859
TC	23	C = 0.140
CC	2	
rs8187722		
AA	65	A = 0.781
AC	45	C = 0.218

**TABLE 5:** Relation of the study parameters to SNP rs12943590 genotypes.

rs12943590	≤6.5 (*n* = 39; 26.17%)			*p*	>6.5 (*n* = 111, 73.82%)			*p*
	GG	GA	AA		GG	GA	AA	
FBG (mg/dL)	135.88 ± 11.30	135.33 ± 18.61	180.00 ± 38.21	0.373	278.38 ± 22.91	244.55 ± 49.19	255.80 ± 55.60	0.833
HbA1C (%)	6.08 ± 0.08	5.80 ± 0.23	6.37 ± 0.17	0.270	9.42 ± 0.49	9.22 ± 0.69	9.98 ± 0.71	0.462
IN (μU/L)	8.41 ± 3.52	5.53 ± 2.08	7.13 ± 2.100	0.812	9.57 ± 2.50	0.85 ± 0.51	3.29 ± 0.85	0.265
IR	49.61 ± 20.96	34.32 ± 13.68	58.01 ± 18.57	0.874	15.36 ± 32.39	15.02 ± 13.78	44.94 ± 15.02	0.300
IS	1.19 ± 0.17	1.22 ± 0.22	1.26 ± 0.11	0.757	1.16 ± 0.11	1.20 ± 0.03	0.81 ± 0.20	0.314
BMI (kg/m^2^)	28.56 ± 1.67	28.09 ± 1.42	28.79 ± 2.48	0.734	28.90 ± 1.10	1.97 ± 1.30	27.36 ± 1.97	0.528

Abbreviations: BMI, body mass index; FBG, fasting blood glucose; HbA1c, glycated haemoglobin; IN, serum insulin; IR, insulin resistant; IS, insulin sensitivity.

As shown in Table 6, regarding the rs8187722 gene, the AC allele showed the lowest mean of HbA1c and HOMA‐IR in good and poor responders but was statistically nonsignificant.

**TABLE 6:** Relation of the study parameters to SNP rs8187722.

rs8187722	≤6.5			*p*	>6.5			*p*
	CC	AC	AA		CC	AC	AA	
FBG (mg/dL)	ND	127.00 ± 5.26	149.10 ± 19.26	0.343	162.00 ± 29.00	145.80 ± 18.35	250.00 ± 13.33	0.442
HbA1C (%)		5.11 ± 0.11	6.93 ± 0.11	0.279	8.19 ± 0.90	8.38 ± 0.39	9.93 ± 0.33	0.383
IN (μU/L)		6.87 ± 1.84	5.76 ± 1.13	0.592	9.85 ± 1.84	6.87 ± 1.03	10.71 ± 2.63	0.400
IR		33.17 ± 10.42	35.90 ± 7.30	0.793	76.97 ± 10.89	74.50 ± 12.28	116.53 ± 28.61	0.375
IS		1.19 ± 0.08	1.22 ± 0.12	0.299	1.1 s1 ± 0.519	1.26 ± 0.04	1.17 ± 0.08	0.180
BMI (kg/m^2^)		28.36 ± 1.19	27.68 ± 0.83	0.631	30.87 ± 1.10	27.77 ± 0.73	27.12 ± 0.57	0.365

Abbreviations: BMI, body mass index; FBG, fasting blood glucose; HbA1c, glycated haemoglobin; IN, serum insulin; IR, insulin resistant; IS, insulin sensitivity; ND, nondetected.

As shown in Table 7, the rs12194182 genotypes showed no statistically significant differences between good and poor responders. In good and poor responders, the TC allele showed a lower mean of HbA1c, FBS and HOMA‐IR than TT and CC but was statistically nonsignificant.

**TABLE 7:** Relation of the study parameters to SNP rs12194182 genotypes.

rs12194182	≤6.5			*p*	>6.5			*p*
	CC	TC	TT		CC	TC	TT	
FBG (mg/dL)	ND	131.71 ± 28.08	137.31 ± 15.78	0.602	236.00 ± 21.00	214.56 ± 21.38	261.76 ± 13.69	0.265
HbA1C (%)		6.32 ± 0.14	6.51 ± 0.10	0.107	10.50 ± 0.01	10.07 ± 0.67	10.44 ± 0.30	0.800
IN (μU/L)		4.73 ± 1.46	9.47 ± 3.34	0.409	1.90 ± 1.55	1.60 ± 4.86	10.96 ± 2.03	0.680
IR		33.18 ± 11.52	55.92 ± 23.28	0.570	121.96 ± 11.17	121.71 ± 45.90	125.79 ± 23.96	0.618
IS		1.94 ± 0.19	1.12 ± 0.137	0.493	1.11 ± 0.48	1.21 ± 0.14	1.08 ± 0.07	0.558
BMI (kg/m^2^)		25.12 ± 1.28	27.74 ± 0.81	0.139	26.74 ± 1.39	26.55 ± 1.13	29.34 ± 1.19	0.829

Abbreviations: BMI, body mass index; HbA1c, glycated haemoglobin; IN, serum insulin; IR, insulin resistant; IS, insulin sensitivity; ND, nondetected.

## 4 Discussion

Diabetes mellitus has emerged as a significant global health concern, with projections indicating that approximately 1 in 12 individuals will be afflicted by the disease in the future. According to the World Health Organization's findings, diabetes mellitus is expected to rank as the seventh leading cause of death worldwide by the year 2030 [17].

Effective control of diabetes is crucial for individuals with the condition because it is closely associated with a wide array of complications, ranging from cardiovascular diseases such as heart attacks and strokes to microvascular issues like diabetic retinopathy and nephropathy, which can ultimately lead to end‐stage renal failure [18].

In our investigation, it was established that 73.82% of patients exhibited inadequate glycaemic control, characterised by an (HbA1c > 6.5%). This proportion closely resembles findings from other research, such as the study by Kassahun, Eshetie, and Gesesew conducted in 2016, which indicated that over two thirds (70.9%) of the 325 participants in Ethiopia experienced poor glycaemic control [19]. In addition, this percentage was higher than the percentages found in Jordanian patients with T2DM (60.8%), which were poorly controlled [20].

In the current research, there was no statistically significant difference between patients who respond well to treatment and those who do not when considering factors like anthropometric data and the most sociodemographic variables. However, differences were observed in patients' medical histories and the duration of their disease, which did impact their response to treatment.

This is consistent with a study conducted in Iran, which involved 103 female diabetic patients classified into controlled and uncontrolled groups on the basis of HbA1c levels. In this Iranian study, the univariate analysis revealed no significant disparities in demographic and anthropometric data between the controlled and uncontrolled groups. Nevertheless, the multivariate analysis identified waist circumference as a significant predictor of elevated HbA1c levels [21].

In our study, we assessed the impact of a promoter variant in MATE2 (SLC47A2 rs12943590) and OCT3 (SLC22A3 rs12194182 and rs8187722) on the variation in metformin response. When we examined the study parameters on the basis of clinical response to metformin according to MATE2 (SLC47A2 rs12943590) and OCT3 (SLC22A3 rs8187722 and rs12194182) genotypes, our results showed no significant differences between good and poor responders with respect to the study parameters. In the present study, SLC47A2 rs12943590 was represented by GG, AA and AG genotypes, with the major allele GG (*n* = 25) and the minor allele AA (*n* = 8), so G>A with allele frequencies of 0.677 and 0.322, respectively The AG genotype of SLC47A2 rs12943590 correlated with a lower level of HOMA‐IR, HbA1c and FBG than AA and GG but was statistically nonsignificant.

This agrees with a study in 2022 conducted by Chen and colleagues in Chaoshan, China, involving 93 patients with T2DM. They found a noteworthy association. Specifically, they observed a significant correlation between individuals with the AG genotype of the rs12943590 polymorphism of the SLC47A2 gene and a more substantial reduction in HOMA‐IR. Additionally, they explored second‐order interactions, particularly between a 30 g dose of metformin and the SLC47A2 rs12943590 genotype, which had a notable impact on fasting insulin levels in patients with T2DM. However, it's important to note that other interaction models did not yield statistically significant results [22].

Moreover, Phani et al., in their 2018 study, reported a significant connection between two genetic variations, SLC22A2 rs316019 and SLC47A2 rs12943590, and the response to metformin treatment. This association was observed in both codominant and dominant genetic models. Notably, individuals with the SLC22A2 rs316019 GG genotype and the SLC47A2 rs12943590 GA genotype exhibited a more pronounced mean change in HbA1c levels [23].

Furthermore, an Egyptian study highlighted a significant difference in the distribution of the MATE2 genotype between healthy individuals and those with T2DM. Among the patients with diabetes, the GG genotype was the most prevalent (54%). This finding suggests that genetic variations in MATE2 might influence the action of metformin in recently diagnosed Egyptian patients with T2DM [24]. Whereas in the 2020 study conducted by Xhakaza and colleagues in the Nguni population of South Africa, they uncovered an interesting association. Specifically, they found that individuals with the GA genotype of the SLC47A2 rs12943590 polymorphism had a reduced response to metformin therapy even after making corrections. The odds ratio for this association was 2.29, with a 95% confidence interval of 1.01–5.21, and the *p* value was 0.01 [25].

The present study, involving 150 subjects, found a noteworthy relationship concerning the rs12194182 SNP of the SLC22A3 gene. Specifically, individuals with the TC genotype exhibited the lowest mean levels of HbA1c, HOMA‐IR and FBG. In contrast, those with the CC and TT genotypes showed higher levels, although these differences were not statistically significant.

These findings align with another study that investigated the impact of genetic polymorphisms in the SLC22A1, SLC22A2 and SLC22A3 genes on metformin pharmacogenetics. This study, conducted with 212 Jordanian patients diagnosed with T2DM, revealed a significant association (*p* < 0.05) between the rs12194182 SNP variant of the SLC22A3 gene and lower mean HbA1c levels. This association was particularly pronounced in patients with the CC genotype [26].

However, it is worth noting that a study involving healthy male Caucasians and examining SLC22A3 SNPs, including rs12194182, rs2292334, rs2504927 and rs3123634, did not find any association with metformin's effects [27].

The current results indicated that individuals with the AG allele of the SLC22A3 rs8187722 variant had lower mean levels of HbA1c and HOMA‐IR compared to those with the AA and GG alleles, although this difference did not reach statistical significance.

These findings are in line with a previous study involving 26 healthy Jordanian volunteers who were taking a daily dose of metformin (1000 mg). In that study, individuals with the SLC22A3 rs8187722 variant had significantly higher metformin *C*_max_ and AUC values compared to those with the wild‐type SLC22A3 genotype. However, there was no significant impact on T½ and Kel. Furthermore, volunteers with the heterozygote SLC22A3 rs2292334 variant exhibited significantly higher metformin Cmax and AUC values and lower Kel values compared to individuals with the wild‐type SLC22A3 genotype, with a *p* < 0.05. These findings suggest that both the SLC22A3 rs8187722 and rs2292334 genetic variants have an influence on metformin pharmacokinetics in a clinical sample of Jordanians [14].

## 5 Conclusion

Variations in genes SLC22A3 and SLC47A2 did not have a significant role in the response of patients with T2DM to metformin (1000 mg/day). It is important to note that several factors contribute to the variation in metformin responses among different populations worldwide. These include ethnicity, gene–gene interactions, and the relationship between genetic factors and the disease itself. These variables can influence the pharmacokinetic and pharmacodynamic properties of the drug, impacting both its effectiveness and potential side effects.

### 5.1 Limitations of the Study

The results of this study might be affected by several limitations. First, the sample size was small, making it difficult to generalise the findings. The small sample size hinders the generalizability of the findings, as they may not accurately represent the broader population. Second, the time limitation of the study may affect the results of these three SNPs on metformin's therapeutic effectiveness.

## Author Contributions

**Alaa Abd Al‐Hussain Naem:** Data curation (equal); Investigation (equal); Methodology (equal). **Mona N. Al‐Terehi:** Data curation (equal); Investigation (equal); Methodology (equal); Validation (equal); Visualization (equal). **Fadhaa Abdulameer Ghafil:** Software (equal); Validation (equal); Visualization (equal); Writing – original draft (equal). **Farid S. Ataya:** Project administration (equal); Resources (equal); Software (equal). **Gaber El‐Saber Batiha:** Project administration (equal); Resources (equal); Software (equal). **Athanasios Alexiou:** Conceptualization (equal); Funding acquisition (equal); Project administration (equal); Resources (equal); Supervision (equal). **Marios Papadakis:** Conceptualization (equal); Funding acquisition (equal); Project administration (equal); Resources (equal); Supervision (equal). **Nermeen N. Welson:** Writing – review and editing (lead). **Najah R. Hadi:** Formal analysis (equal); Validation (equal); Visualization (equal); Writing – original draft (equal).

## Conflicts of Interest

The authors declare no conflicts of interest.

## Data Availability

Data are available with corresponding author upon reasonable request.

## References

[1] K. A. Kirtland , P. Cho , and L. S. Geiss , “Diabetes Among Asians and Native Hawaiians or Other Pacific Islanders—United States, 2011–2014,” Morbidity and Mortality Weekly Report 64, no. 45 (2015): 1261–1266. 26583766 10.15585/mmwr.mm6445a2

[2] D. Mozaffarian , E. J. Benjamin , A. S. Go , et al., “Heart Disease and Stroke Statistics—2015 Update: A Report From the American Heart Association,” Circulation 131, no. 4 (2015): e29–e322. 25520374 10.1161/CIR.0000000000000152

[3] G. Rena , E. R. Pearson , and K. Sakamoto , “Molecular Mechanism of Action of Metformin: Old or New Insights?” Diabetologia 56 (2013): 1898–1906. 23835523 10.1007/s00125-013-2991-0 PMC3737434

[4] G. Zhou , R. Myers , Y. Li , et al., “Role of AMP‐Activated Protein Kinase in Mechanism of Metformin Action,” The Journal of Clinical Investigation 108, no. 8 (2001): 1167–1174. 11602624 10.1172/JCI13505 PMC209533

[5] E. Ferrannini , “The Target of Metformin in Type 2 Diabetes,” New England Journal of Medicine 371, no. 16 (2014): 1547–1548. 25317875 10.1056/NEJMcibr1409796

[6] K. Zhou , C. Bellenguez , C. Spencer , et al., “Common Variants Near ATM Are Associated With Glycemic Response to Metformin in Type 2 Diabetes,” Nature Genetics 43, no. 2 (2011): 117–120. 21186350 10.1038/ng.735 PMC3030919

[7] K. Zhou , L. Donnelly , J. Yang , et al., “Heritability of Variation in Glycaemic Response to Metformin: A Genome‐Wide Complex Trait Analysis,” The Lancet Diabetes & Endocrinology 2, no. 6 (2014): 481–487. 24731673 10.1016/S2213-8587(14)70050-6 PMC4038749

[8] M. L. Reitman and E. E. Schadt , “Pharmacogenetics of Metformin Response: A Step in the Path Toward Personalized Medicine,” The Journal of Clinical Investigation 117, no. 5 (2007): 1226–1229. 17476355 10.1172/JCI32133 PMC1857273

[9] Y. Shu , S. A. Sheardown , C. Brown , et al., “Effect of Genetic Variation in the Organic Cation Transporter 1 (OCT1) on Metformin Action,” The Journal of Clinical Investigation 117, no. 5 (2007): 1422–1431, 10.1172/JCI30558. 17476361 PMC1857259

[10] M. L. Becker , L. E. Visser , R. H. van Schaik , A. Hofman , A. G. Uitterlinden , and B. H. Stricker , “Genetic Variation in the Multidrug and Toxin Extrusion 1 Transporter Protein Influences the Glucose‐Lowering Effect of Metformin in Patients With Diabetes: A Preliminary Study,” Diabetes 58, no. 3 (2009): 745–749, 10.2337/db08-1028. 19228809 PMC2646075

[11] D. A. Trégouët , I. R. König , J. Erdmann , et al., “Genome‐Wide Haplotype Association Study Identifies the SLC22A3‐LPAL2‐LPA Gene Cluster as a Risk Locus for Coronary Artery Disease,” Nature Genetics 41, no. 3 (2009): 283–285, 10.1038/ng.314. 19198611

[12] S. A. Tomlins , R. Mehra , D. R. Rhodes , et al., “Integrative Molecular Concept Modeling of Prostate Cancer Progression,” Nature Genetics 39, no. 1 (2007): 41–51, 10.1038/ng1935. 17173048

[13] A. T. Nies , K. Damme , S. Kruck , E. Schaeffeler , and M. Schwab , “Structure and Function of Multidrug and Toxin Extrusion Proteins (MATEs) and Their Relevance to Drug Therapy and Personalized Medicine,” Archives of Toxicology 90, no. 7 (2016): 1555–1584, 10.1007/s00204-016-1728-5. 27165417

[14] N. Hakooz , Y. B. Jarrar , M. Zihlif , A. Imraish , S. Hamed , and T. Arafat , “Effects of the Genetic Variants of Organic Cation Transporters 1 and 3 on the Pharmacokinetics of Metformin in Jordanians,” Drug Metabolism and Personalized Therapy 32, no. 3 (2017): 157–162. 28862982 10.1515/dmpt-2017-0019

[15] A. Katz , S. S. Nambi , K. Mather , et al., “Quantitative Insulin Sensitivity Check Index: A Simple, Accurate Method for Assessing Insulin Sensitivity in Humans,” The Journal of Clinical Endocrinology & Metabolism 85, no. 7 (2000): 2402–2410. 10902785 10.1210/jcem.85.7.6661

[16] B. Antuna‐Puente , M. Faraj , A. D. Karelis , et al., “HOMA or QUICKI: Is It Useful to Test the Reproducibility of Formulas?” Diabetes & Metabolism 34, no. 3 (2008): 294–296. 18468934 10.1016/j.diabet.2008.02.001

[17] J. E. Shaw , R. A. Sicree , and P. Z. Zimmet , “Global Estimates of the Prevalence of Diabetes for 2010 and 2030,” Diabetes Research and Clinical Practice 87, no. 1 (2010): 4–14. 19896746 10.1016/j.diabres.2009.10.007

[18] M. Mohamed and D.‐A. S. Group , “An Audit on Diabetes Management in Asian Patients Treated by Specialists: The Diabcare‐Asia 1998 and 2003 Studies,” Current Medical Research and Opinion 24, no. 2 (2008): 507–514. 18184454 10.1185/030079908x261131

[19] T. Kassahun , T. Eshetie , and H. Gesesew , “Factors Associated With Glycemic Control Among Adult Patients With Type 2 Diabetes Mellitus: A Cross‐Sectional Survey in Ethiopia,” BMC Research Notes 9, no. 1 (2016): 1–6. 26861243 10.1186/s13104-016-1896-7 PMC4748519

[20] L. N. Al‐Eitan , A. M. Nassar , N. A. Saadeh , and B. A. Almomani , “Evaluation of Glycemic Control, Lifestyle and Clinical Characteristics in Patients With Type 2 Diabetes Treated at King Abdullah University Hospital in Jordan,” Canadian Journal of Diabetes 40, no. 6 (2016): 496–502. 27212046 10.1016/j.jcjd.2016.04.009

[21] Z. Ghazanfari , S. Niknami , F. Ghofranipour , B. Larijani , H. Agha‐Alinejad , and A. Montazeri , “Determinants of Glycemic Control in Female Diabetic Patients: A Study from Iran,” Lipids in Health and Disease 9, no. 1 (2010): 1–5. 20701805 10.1186/1476-511X-9-83 PMC2931513

[22] P. Chen , Y. Cao , S. Chen , Z. Liu , S. Chen , and Y. Guo , “Association of SLC22A1, SLC22A2, SLC47A1, and SLC47A2 Polymorphisms With Metformin Efficacy in Type 2 Diabetic Patients,” Biomedicine 10, no. 10 (2022): 2546. 10.3390/biomedicines10102546 PMC9599747 36289808

[23] N. M. Phani , M. Vohra , A. Kakar , et al., “Implication of Critical Pharmacokinetic Gene Variants on Therapeutic Response to Metformin in Type 2 Diabetes,” Pharmacogenomics 19, no. 11 (2018): 905–911. 29914345 10.2217/pgs-2018-0041

[24] G. Mostafa‐Hedeab , A. A. Mohamed , G. Thabet , D. Sabry , R. F. Salam , and M. E. Hassen , “Effect of MATE 1, MATE 2 and OCT1 Single Nucleotide Polymorphisms on Metformin Action in Recently Diagnosed Egyptian Type‐2 Diabetic Patients,” Biomedical and Pharmacology Journal 11, no. 1 (2018): 149–157.

[25] L. Xhakaza , Z. Abrahams‐October , B. Pearce , et al., “Evaluation of the Suitability of 19 Pharmacogenomics Biomarkers for Individualized Metformin Therapy for Type 2 Diabetes Patients,” Drug Metabolism and Personalized Therapy 35, no. 2 (2020): 20200111. 10.1515/dmpt-2020-0111 32681778

[26] L. N. Al‐Eitan , B. A. Almomani , A. M. Nassar , B. Z. Elsaqa , and N. A. Saadeh , “Metformin Pharmacogenetics: Effects of SLC22A1, SLC22A2, and SLC22A3 Polymorphisms on Glycemic Control and HbA1c Levels,” Journal of Personalized Medicine 9, no. 1 (2019): 17. 30934600 10.3390/jpm9010017 PMC6462993

[27] M. V. Tzvetkov , S. V. Vormfelde , D. Balen , et al., “The Effects of Genetic Polymorphisms in the Organic Cation Transporters OCT1, OCT2, and OCT3 on the Renal Clearance of Metformin,” Clinical Pharmacology & Therapeutics 86, no. 3 (2009): 299–306. 19536068 10.1038/clpt.2009.92

